# Genomic Variation and Host Interaction among *Pseudomonas syringae* pv. *actinidiae* Strains in *Actinidia chinensis* ‘Hongyang’

**DOI:** 10.3390/ijms23179743

**Published:** 2022-08-28

**Authors:** Yu Zhou, Shengxiong Huang, Wei Tang, Zhongqiu Wu, Siqi Sun, Yaqiong Qiu, Hongtao Wang, Xue Chen, Xiaofeng Tang, Fangming Xiao, Yongsheng Liu, Xiangli Niu

**Affiliations:** 1 School of Food and Biological Engineering, Hefei University of Technology, Hefei 230601, China; 2 School of Horticulture, Anhui Agricultural University, Hefei 230036, China; 3 Anhui Jiaotianxiang Biological Technology Co., Ltd., Xuancheng 242099, China; 4 Department of Plant Sciences, University of Idaho, Moscow, ID 83844, USA

**Keywords:** *Pseudomonas syringae* pv. *actinidiae*, *Actinidia chinensis*, pathogenicity, genome analysis, transcriptome analysis, plant-pathogen interactions

## Abstract

Kiwifruit bacterial canker is a recent epidemic disease caused by *Pseudomonas syringae* pv. *actinidiae* (*Psa*), which has undergone worldwide expansion in a short time and resulted in significant economic losses. ‘Hongyang’ (*Actinidia chinensis*), a widely grown cultivar because of its health-beneficial nutrients and appreciated red-centered inner pericarp, is highly sensitive to *Psa*. In this work, ten *Psa* strains were isolated from ‘Hongyang’ and sequenced for genome analysis. The results indicated divergences in pathogenicity and pathogenic-related genes among the *Psa* strains. Significantly, the interruption at the 596 bp of *HrpR* in two low-pathogenicity strains reemphasized this gene, expressing a transcriptional regulator for the effector secretion system, as an important pathogenicity-associated locus of *Psa*. The transcriptome analysis of ‘Hongyang’ infected with different *Psa* strains was performed by RNA-seq of stem tissues locally (at the inoculation site) and systemically. *Psa* infection re-programmed the host genes expression, and the susceptibility to *Psa* might be attributed to the down-regulation of several genes involved in plant-pathogen interactions, especially calcium signaling transduction, as well as fatty acid elongation. This suppression was found in both low- and high-pathogenicity *Psa* inoculated tissues, but the effect was stronger with more virulent strains. Taken together, the divergences of *P. syringae* pv. *actinidiae* in pathogenicity, genome, and resulting transcriptomic response of *A*. *chinensis* provide insights into unraveling the molecular mechanism of *Psa*-kiwifruit interactions and resistance improvement in the kiwifruit crop.

## 1. Introduction

Kiwifruit (*Actinidia* spp.) is one of the most recently domesticated fruit crops, starting in the 1930s. After *Actinidia deliciosa* ‘Hayward’ was developed in New Zealand in the 1980s, the commercial cultivation of kiwifruit became popular due to its nutritional and health attributes [1]. The planting area continuously increased for green-fleshed ‘Hayward’ and subsequently yellow-fleshed *A*. *chinensis* ‘Hort16A’, ‘Jin Tao’, and red-fleshed ‘Hongyang’ in New Zealand and other countries such as China and Italy [2,3,4]. Unfortunately, the intensive cultivation of the clonally propagated kiwifruit population also provided opportunities for the emergence (1984) and re-emergence (2008) of the pandemic pathogen of *Pseudomonas syringae* pv. *actinidiae* (*Psa*), causing bacterial canker disease and serious economic losses of kiwifruit worldwide [5,6,7]. However, the mechanisms underlying the pathogenicity of *Psa* and host plant response to the pathogen remain unclear.

When first reported in 1984 in Japan and China, and then isolated in 1992 in Italy, *Psa* was found to cause canker symptoms, leaf necrosis, twig die-back, trunk cankers, and plant death on *A*. *deliciosa* in Asia. This disease did not then seriously occur in European orchards. But the sudden re-emergence of the epidemic as a more destructive infectious disease during 2008–2011 has heavily affected almost all cultivars of this crop worldwide [5,7,8]. Coincident with the hypothesis for other agricultural pathovars to be evolved from commensal or pathogenic microorganisms colonizing wild relatives of domesticated crops, it was presumed that a related pathovar of *P*. *syringae* pv. *theae* or *P*. *amygdali* contributed as a donor bacterium to transfer host adaptation and pathogenicity to *Actinidia* species and thus the emergence of *P*. *syringae* pv. *actinidiae* [9,10]. Similar to other *P*. *syringae* pathovars, *Psa* is also a rod-shaped Gram-negative bacterium with polar flagella. This recently evolved new member attacks its host by penetrating almost all kinds of kiwifruit tissues via stomata, lenticel, abscission scars, pruning cuts, and then spreading systemically through colonization in xylem and phloem vessels [11,12,13]. So far, five *Psa* populations (biovar 1, 2, 3, 5, and 6) have been identified [14]. Compared with biovar 1 (Psa1, reported in Japan, 1984–1989; Italy, 1992) and biovar 2 (Psa2, South Korea, 1988), *Psa* biovar 3 (Psa3, responsible for the global epidemic of 2008) represented an independent phylogenetic lineage with enhanced epidemic potentials by gain and/or loss of virulence/avirulence factors and ecological fitness related genes [8,9,10], in which various mobile elements, distributed throughout the genome, were considered as major drivers of this evolution [10,15]. After it was introduced into other European growing areas from Italy in 2008, Psa3 has established heterogeneous populations with increased transposons in the bacterial genome [14,16]. The abundant genomic diversity co-existing within Psa3 and among *Psa* biovars maintains reservoirs to allow for novel pathovar and future epidemics, as happened with Psa3. Fortunately, the bacterial variants generated from genomic diversity also provide approaches to unraveling the pathogenicity formation of *Psa* [16,17]. Accordingly, kiwifruit (domesticated since the 1930s) and coevolved pathogen *Psa* (firstly reported in the 1980s) has become a rare case for tracking crop disease due to the clearly documented domestication and epidemic history [2,4,7].

China is the center of origin and distribution of the *Actinidia* genus [4]. After the epidemic of 2008, canker-causing *Psa* strains have been isolated from diverse kiwifruit cultivation areas and regions of wild *Actinidia* species in China [17,18,19]. All isolates belonged to the Psa3 group, with broad diversity, suggesting regional transmission within China after the pandemic. In our previous work, the analysis of *A. chinensis* ‘Hongyang’ identified putative genes involved in PAMP-triggered immunity and effector-triggered immunity in the genome [3]. However, ‘Hongyang’ is highly susceptible to *Psa*, as manifested by field surveys and in vitro assays [5,6,20]. In the present work, multiple *Psa* strains were isolated from canker diseased ‘Hongyang’, and 10 isolates with differential pathogenicity were sequenced to determine their genetic differences. Furtherly, the comparative transcriptomic analysis of the ‘Hongyang’ response to strains with different pathogenicity was performed by RNA-seq to illustrate the molecular mechanism of kiwifruit-canker pathogen interactions.

## 2. Materials and Methods

### 2.1. Isolation of P. syringae pv. actinidiae

The twigs and canes of kiwifruit with canker symptoms were collected from orchards in Beichuan, Dujiangyan, Guangyuan, Muchuan, Pengzhou, Yaan, Yibin in the Sichuan Province, Hefei in the Anhui Province, and Enshi in the of Hubei Province. Bacteria isolation was performed by homogenizing sampled tissues in 10 mM MgCl_2_ and plating the homogenate on King’s B (KB, 10 mL/L glycerol, 20 g/L tryptone, 1.5 g/L K_2_HPO_4_, 1.5 g/L MgSO_4_) agar plates for incubation at 25 °C. The *Psa*-like single colonies were re-streaked for further purification and then used for specific PCR identification by amplifying a portion of the 16S-23S rDNA inter transcribed spacer region (MT423619) or *ompP1* gene (*outer membrane protein P1*, MH084697) of *Psa* [21,22]. After sequencing verification of the PCR products, the *Psa* isolates were stored at −80 °C. The ten *Psa* strains, ScDjyH (2016), AhHfH (2017), ScBcH (2017), ScGyH1 (2017), ScGyH2 (2017), ScMcH (2017), ScPzH (2017), ScYaH (2018), ScYbH (2018) and HbEsH (2019), isolated from ‘Hongyang’ were used for further assays.

### 2.2. Plants Materials and Growth Conditions

Wild-type tobacco (*Nicotiana benthamiana* and *Nicotiana tabacum*) plants were grown in the artificial climate chamber (25 °C, 16 h light/8 h dark). The 4- to 5-week-old tobacco plants were used for hypersensitivity response (HR) elicitation. The tissue-cultured ‘Hongyang’ were grown in a growth chamber under normal conditions (25 °C, 16 h light/8 h dark, 60% relative humidity). After an inoculation of *Psa*, the growth conditions were modified (16 °C, 16 h light/8 h dark, 80–90% relative humidity). ‘Hongyang’ explants (2–3 cm tall) or plantlets (5–7 fully expanded leaves, approximately 30 cm tall) were used for *Psa* inoculation and transcriptomic analyses.

### 2.3. Psa Inoculation

The *Psa* bacterial suspensions (10^7^~5 × 10^8^ CFU) were infiltrated into 4- to 5-week-old tobacco leaves to elicit a hypersensitivity response. Prf^D1416V^, an auto-active resistance Prf protein triggering HR cell death [23], or 10 mM MgCl_2_ was injected as a positive or negative control, respectively. For kiwifruit, the tissue-cultured ‘Hongyang’ explants were used for flood-inoculation as described [24] with minor modifications. Forty milliliters of bacterial suspension (5 × 10^8^ CFU) in sterile distilled water containing 0.025% (*v*/*v*) silwet L-77 was dispensed onto a medium plate with ‘Hongyang’ explants for a 3-min incubation. After the bacterial suspension was removed, inoculated plants (without washing after inoculation) were grown at 16 °C for 12 days. For the transcriptomic analysis, the stem of the ‘Hongyang’ plantlet was surface sterilized and shaved to form a wound (1.0 cm) for inoculation of 10 μL *Psa* suspension (10^9^ CFU). Ten microliters of 10 mM MgCl_2_ were used as mock inoculation. The inoculated ‘Hongyang’ were grown in the chamber with 90% humidity for the first 24 h and then with 80% humidity at 16 °C. At 14 dpi (days post-inoculation), three biological replicates of stem tissues both at inoculation sites (local) and 2 cm above the inoculation sites (systemic) of each plantlet were respectively sampled for *Psa* colony counting and transcriptomic sequencing. All *Psa*-inoculation experiments were performed three times with at least three biological replicates per experiment. The inoculated triplicates of ‘Hongyang’ explants or plantlet stems were taken for *Psa* colony counting. One-way ANOVA (analysis of variance) was used for statistical analysis, and statistical differences were indicated by different letters at *p* < 0.05.

### 2.4. Genome Analysis of Psa

Genomic DNA of the *Psa* strains, ScDjyH, AhHfH, ScBcH, ScGyH1, ScGyH2, ScMcH, ScPzH, ScYaH, ScYbH, HbEsH, were extracted using Wizard Genomic DNA Purification Kit (Promega Madison, WI, USA) and quantified with TBS-380 fluorometer. Qualified DNA (1 μg, OD_260/280_ = 1.8~2.0) for each strain was sheared to 400–500 bp fragments using a Covaris M220 Focused Acoustic Shearer to construct a library using NEXTflex Rapid DNA-Seq Kit (Bioo Scientific, Austin, TX, USA) for paired-end sequencing on the Illumina HiSeq X Ten platform. The low-quality raw reads were removed by quality trimming to form clean data for genome assembly using SOAPdenovo2 and CDS prediction by Glimmer, and then for the analysis of carbohydrate-active enzyme, secondary metabolism cluster, transporter, drug-resistance, CRISPR (Clustered Regularly Interspaced Short Palindromic Repeats)-Cas related genes, as well as the virulence factor, pathogen-host interaction, secretion system, secretion protein, genomic island, prophage, core, and specific genes among the *Psa* strains. The raw sequence data were deposited in the BioProject database of NCBI (http://www.ncbi.nlm.nih.gov) with accessions PRJNA763857 and PRJNA794320. The insertions and variations in *HrpR* and type three effectors were verified by gene cloning and sequencing.

### 2.5. Transcriptomic Analysis of Inoculated ‘Hongyang’

Total RNA was extracted from the sampled ‘Hongyang’ tissues using TRIzol (Invitrogen, Carlsbad, California, USA) and genomic DNA removed by Dnase I (TaKara, Beijing, China). RNA was detected with 2100 Bioanalyser, and 1 μg of qualified RNA (28S:18S ≥ 1.0, OD_260/230_ ≥ 2.0, OD_260/280_ = 1.8~2.2, RIN ≥ 6.5) for each sample was used to construct RNA-seq library by a TruSeq preparation Kit (Illumina, San Diego, CA, USA), and used for sequencing with the Illumina HiSeq X Ten. The raw reads were trimmed by Sickle and SeqPrep tools, and the resulting clean reads were aligned according to the reference genome of *A*. *chinensis* by TopHat. The TPM (Transcripts Per Million reads) method was used for expression level calculation, and DESeq2 was used for differential expression analysis. Then the KEGG metabolic pathway enrichment of differentially expressed genes (DEGs) was analyzed by KOBAS, and the WGCNA (Weighted Gene Co-Expression Network Analysis) was performed for all of the 18 RNA-seq samples. The raw sequence data were deposited in the BioProject database of NCBI with accessions PRJNA793762 and PRJNA794862.

### 2.6. Real-Time Quantitative PCR Analysis

Total RNA was isolated from *Psa*-inoculated ‘Hongyang’ stems. cDNA was synthesized using TransScript One-Step gDNA Removal and cDNA Synthesis SuperMix (Transgen Biotech, Beijing, China). Real-time quantitative PCR was performed on the Bio-Rad CFX 96 Manager (Bio-Rad, Hercules, CA, USA) using PerfectStart Green qPCR SuperMix (Transgen Biotech, Beijing, China). *AcActin* (EF063572) served as the reference gene [25]. The primers used for RT-qPCR are listed in Supplementary Materials Table S1. Each sample was amplified in triplicate, and gene expression levels were quantified with the 2-Ct method. Statistical significance was determined using Student’s *t*-test (* *p* < 0.05, ** *p* < 0.01).

## 3. Results and Discussion

### 3.1. Difference in Pathogenicity of the Isolated Psa Strains

**Figure 1:**
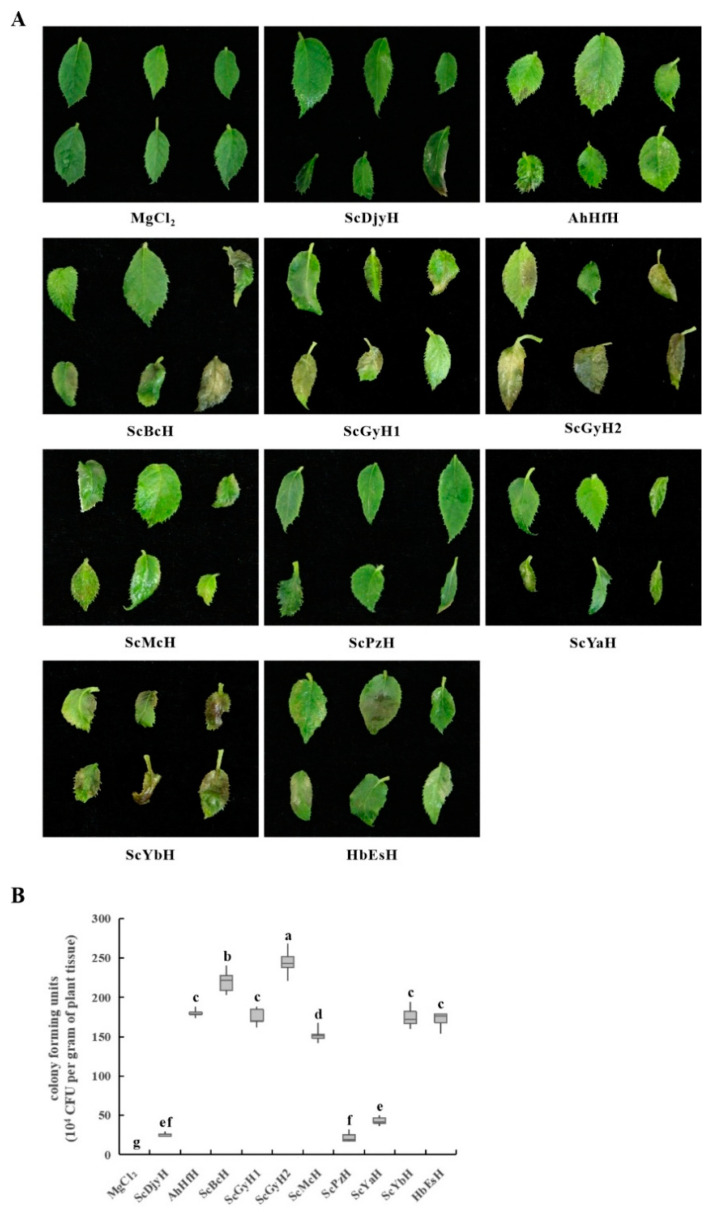
Disease symptoms and bacterial populations in ‘Hongyang’ infected by *Psa* strains. The tissue-cultured ‘Hongyang’ explants were flood inoculated with *Psa* bacterial suspension (5 × 10^8^ CFU), and 10 mM MgCl_2_ was used as a control. Disease symptoms (**A**) and *Psa* populations (**B**) of explant leaves were scored at 12 dpi. Different letters on the boxes indicate statistical differences at *p* < 0.05.

As described in ‘Materials and Methods’, *Psa* strains were isolated from diseased ‘Hongyang’ in the orchards of Sichuan, Anhui, and Hubei during 2016–2019 for pathogenicity determination. The flood-inoculated ‘Hongyang’ explants showed different levels of infection indicative of the pathogenicity of the *Psa* strain used for inoculation (Figure 1A and Figure S1). AhHfH, ScBcH, ScGyH1, ScGyH2, ScMcH, ScYbH, and HbEsH displayed more serious canker disease-related symptoms, specks, and lesions at inoculated leaves than ScDjyH, ScPzH or ScYaH did at 12 dpi, consistent with their significantly increased levels of bacterial colonization (Figure 1B), indicating different degrees of pathogenicity existing in these ten *Psa* strains.

**Figure 2:**
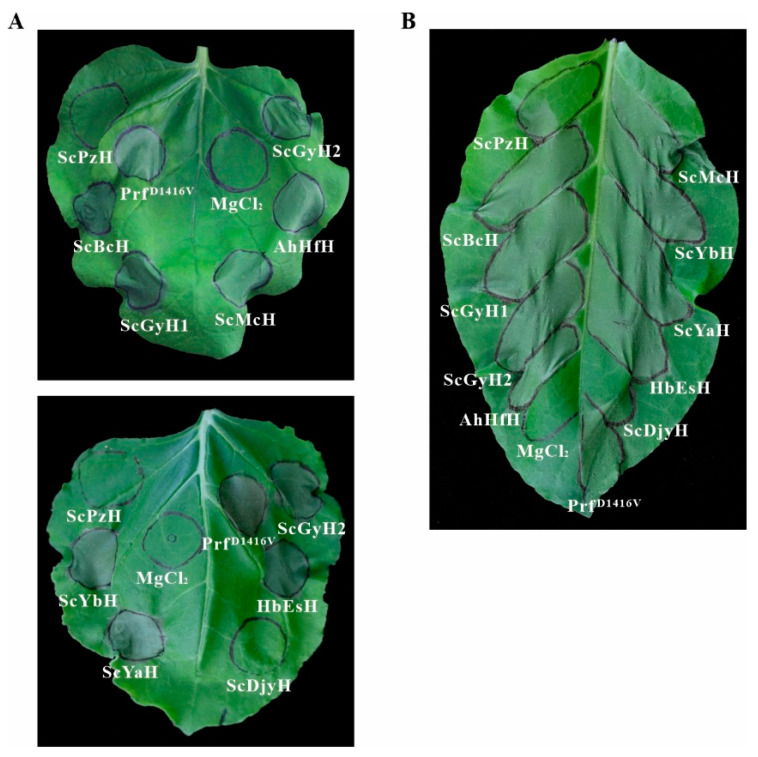
Hypersensitivity response of *N*. *benthamiana* (**A**) and *N*. *tabacum* (**B**) elicited by the *Psa* strains. Leaves of 4-to 5-week-old tobacco plants were infiltrated with *Psa* suspension (10^7^ CFU) for 48 h. Prf^D1416V^ and 10 mM MgCl_2_ were included as a positive and negative control, respectively.

*Psa* can elicit a hypersensitivity response in *N*. *benthamiana* or *N*. *tabacum* leaves [16,19]. For the *Psa* strains tested here, AhHfH, ScBcH, ScGyH1, ScGyH2, ScMcH, ScYaH, ScYbH, and HbEsH could elicit HR after injection into tobacco leaves at 10^7^ CFU for 24–48 h. In comparison, ScDjyH and ScPzH could not trigger any HR reaction (Figure 2) even at the concentration of 5 × 10^8^ CFU for 72 h (Supplementary Materials Figure S2). Additionally, AhHfH, ScBcH, ScGyH1, ScGyH2, ScMcH, and ScPzH were also infiltrated into Arabidopsis leaves at 10^7^ CFU. All tested strains, except ScPzH, caused obvious symptoms of leaf wilting and necrotic spots on leaf surface at 3–4 dpi (Supplementary Materials Figure S3), suggesting the similar infection difference of *Psa* bacteria in *Arabidopsis thaliana*.

Taken together, the ten representative *Psa* strains isolated from ‘Hongyang’ grown in nine orchards in three provinces during 2016–2019 possess distinct pathogenicity, implying their genetic diversity for phenotypic variability.

### 3.2. Genomic Diversity of the Psa Strains

To investigate the genome of *Psa*, AhHfH (2017), ScBcH (2017), ScGyH1 (2017), ScGyH2 (2017), ScMcH (2017), ScPzH (2017), ScDjyH (2016), ScYaH (2018), ScYbH (2018), HbEsH (2019) were subjected for genome sequencing (Supplementary Materials Table S2.1). All the ten *Psa* strains belonged to Psa3 based on genome sequence mapping. The genome size ranged from 6,063,125 bp to 6,264,163 bp, GC content from 58.47% to 58.63%, with 5575 to 6107 annotated coding sequences in the NR database (Table 1). Genes encoding carbohydrate-active enzymes, genes involved in secondary metabolism, transporter, drug-resistance, and CRISPR-Cas system were presented in Supplementary Materials Tables S2.2–S2.6.

**Figure 3:**
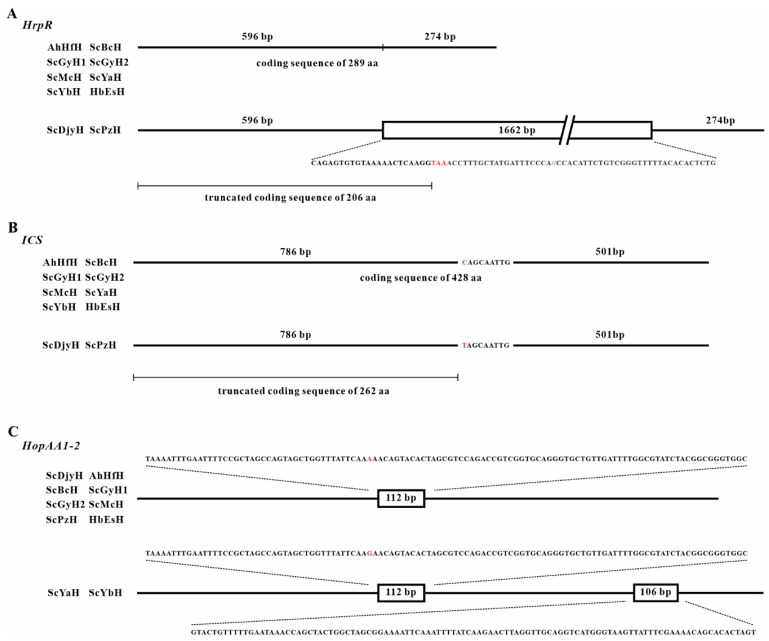
Schematic diagram of *HrpR* (**A**), *ICS* (**B**), and type III effector *HopAA1-2* (**C**). The black lines represent gene structure. The boxes of ‘1662 bp’, ‘112 bp’, and ‘106 bp’ indicate where the mobile elements are inserted. The corresponding insertion sequences are shown by open dots. The stop codon TAA in the insertion of *HrpR*, C/T mutation in *ICS,* and A/G polymorphism in the insertion fragment of *HopAA1-2* are highlighted in red.

The virulence and secretion system-associated genes were also analyzed (Table 1 and Tables S2.7–S2.10), the differences of which may result in varied bacterial pathogenicity among the strains. The low-pathogenicity ScPzH included the least number of genes involved in pathogen-host interaction (955) and secretion system (72). Type IV secretion system-associated genes, VirB8, VirB9, VirB10, VirB11, and VirD4, were not found in ScPzH (Supplementary Materials Table S2.9). The type III secretion system (TTSS) consists of the hrp/hrc cluster and transcriptional regulatory proteins (HrpR, HrpS, HrpL), and the HrpR/S-HrpL-effector cascade controls its function [26,27]. The *HrpR* gene of the two low-pathogenicity strains ScPzH and ScDjyH was interrupted by a 1662 bp insertion at 596 bp, resulting in a presumably truncated protein of 206 aa by fusing a stop codon in the inserted sequence (Figure 3A). This might attenuate the transcription and secretion of type III effectors (TTEs). Moreover, the *ICS* (*isochorismate synthase*) gene in salicylate biosynthesis also indicated a truncated coding sequence in ScPzH and ScDjyH by introducing a mutated stop codon (Figure 3B).

Additionally, genetic variations of effectors were shown among these *Psa* strains. Some effectors were found exclusively in the genome of specific strains; for example, *HopAM1* and helper *HopAK1* were found in AhHfH; *HopAV1* was only found in ScYbH. As for *HopAA1-2*, it was reported that a 106 bp mobile element was inserted in this gene of the *Psa* strain NZ V-13 isolated in New Zealand, resulting in a frameshift [9]. However, in ScYaH and ScYbH, *HopAA1-2* was interrupted twice by the 106 bp Pac_ICE element plus another 112 bp insertion fragment (Figure 3C). This 106 bp mobile DNA element is abundantly distributed in the genome and plasmid of *Psa*. The 112 bp insertion fragment is like a chimera composed half of sequences identical with *Psa* and half of another *Pseudomonas* pathovar, respectively, and inserted as a whole in the genome of some *Psa* strains. More complicatedly, the 112 bp insertion fragment has an A/G polymorphism in the 43rd nucleotide. The former (nucleotide A) was presented in previously sequenced strains, including P155, CRAFRU 14.08, CRAFRU 12.29, NZ-47, NZ-45, ICMP 18708, ICMP 18884, and in strains ScDjyH, AhHfH, ScBcH, ScGyH1, ScGyH2, ScMcH, ScPzH, and HbEsH. The latter (G), presented in ScYaH and ScYbH, was only found in the Chinese *Psa* strain Shaanxi_M228. The combined insertions shown in ScYaH and ScYbH suggest novel genetic variation.

The ten *Psa* strains shared 4848 core genes but possessed various specific genes (Supplementary Materials Figure S4 and Table S2.11), further suggesting their diversity. For instance, a gene encoding an ultraviolet light resistance protein was specifically annotated in ScPzH, and three genes encoding threonine dehydratase, agmatine deiminase, and aliphatic isothiocyanate resistance protein, respectively, were specific for the virulent ScGyH2, ScMcH, HbEsH strains. Moreover, the *Psa* strains possessed various genomic islands and prophages (Supplementary Materials Tables S2.12 and S2.13). Particularly, the low-pathogenicity ScPzH and ScDjyH had one and two islands, respectively, whereas four to ten islands were found in the genomes of the other strains, suggesting different horizontal transfer events have occurred in these *Psa* strains.

The heterogeneity among *Psa* has been found in Europe [16] and China [17,19], and it is the same for the *Psa* presented here. Significantly, rapid gene variations driven by mobile elements still occurred, as exemplified by *HopAA1-2* in ScYaH and ScYbH (Figure 3). Additionally, ICE (integrative conjugative elements)-mediated highly efficient transfer of ICE carriage (both in vitro and in planta) have been found in *Psa* [28]. Undoubtedly, genetic transfer among *Psa* populations would further accelerate its diversity with potential changes in ecological fitness, virulence, and host range. In fact, it has been recently reported that *Psa* can cause leaf spot disease on *Broussonetia papyrifera* (an economic tree species) grown nearby kiwifruit orchards [29], suggesting a possible expansion of the *Psa* host range.

On the other hand, the presence of *Psa* variants also facilitates revealing the underlying pathogenicity-associated loci. Firrao et al. (2018) reported the European *Psa* isolates (CRAFRU 12.50, CRAFRU 12.29) could not elicit HR on tobacco leaves or cause foliar symptoms in kiwifruit, presumably due to the ISPsy36/ISPsy31 insertion in *HrpR* or *HrpS*, interrupting the HrpR/S-HrpL-effector regulatory cascade. A similar case was found in the M227 strain isolated in China: a transposable insertion at the 930 bp upstream of *HrpR* caused reduced pathogenicity by attenuating the expression of TTSS and TTEs [17]. For the other three non-pathogenic strains (S2, G4, G40), the ISPsy36 transposon insertion was found at the 573 bp position of *HrpR* [30]. In the present work, the *HrpR* gene of two low-pathogenicity strains, ScPzH and ScDjyH, was interrupted at 596 bp, resulting in a mutated gene encoding a truncated protein (Figure 3A). In addition, ScPzH was annotated with the least number of secretory system-associated genes. ScPzH and ScDjyH shared unique variations different from the virulent *Psa* strains, suggesting more genetic loci deserve to be investigated.

### 3.3. Transcriptomic Response of ‘Hongyang’ to the Psa with different Pathogenicity

**Figure 4:**
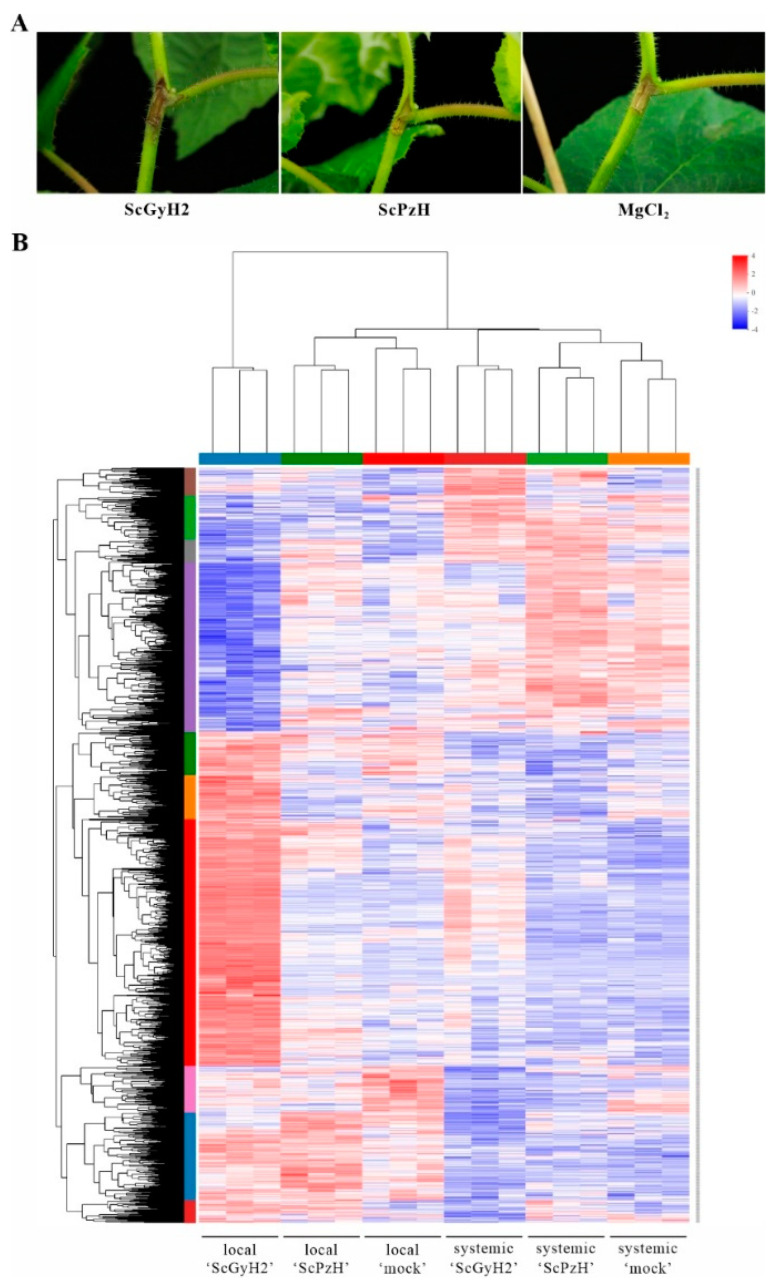
Transcriptomic responses of ‘Hongyang’ to the *Psa* strains ScPzH and ScPzH. (**A**) Disease symptoms of the *Psa* inoculated plantlets. Tissue-cultured ‘Hongyang’ were wound-inoculated with 10^9^ CFU of ScGyH2 (left), ScPzH (middle), or 10 mM MgCl_2_ (right, mock inoculation). All analyses were performed after 14 days. (**B**) Expression heatmap of the unigenes in ScGyH2, ScPzH, or mock-inoculated stem tissues at inoculation sites (local ‘ScGyH2′, local ‘ScPzH, local ‘mock’, respectively) and 2 cm above the inoculation sites (systemic ‘ScGyH2′, systemic ‘ScPzH, systemic ‘mock’). Three biological replicates were included for transcriptomic analysis. The color scale indicates a relative fold-change in expression, where red color shows high expression and blue for low expression.

‘Hongyang’ plantlets were inoculated with the low-pathogenicity ScPzH or high-pathogenicity ScGyH2 strain. The inoculated local (inoculation site) and systemic (2 cm above the inoculation site) stem tissues were collected at 14 dpi (Figure 4A) for transcriptomic analysis. There were no obvious disease symptoms found at the local sites of ScPzH inoculated ‘Hongyang’ (local ‘ScPzH’) and mock group (local ‘mock’) with lower *Psa* bacteria colonization (2 × 10^6^ and 0 CFU, respectively) compared with the disease symptoms and *Psa* scoring (3 × 10^7^ CFU) shown in local ‘ScGyH2′. As summarized in Supplementary Materials Table S3.1, 898,331,894 clean reads for 133,660,934,927 sequencing bases reflected the performance of ‘Hongyang’ to ScPzH and ScGyH2. The local ‘ScPzH’ tissues displayed consistently similar expression patterns as local ‘mock’, contrary to the drastic changes of large amounts of transcripts shown in local ‘ScGyH2′, although local ‘ScGyH2′ and ‘ScPzH’ also shared substantial numbers of up- or down-regulated genes implying common responses against *Psa* (Figure 4B). For systemic tissues, the ‘ScPzH’ group maintained similar expression patterns as ‘mock’. The 14-day infected systemic ‘ScGyH2′ did not reach the levels shown in local ‘ScGyH2′, but a substantial portion of the transcripts indicated up- or down-regulation consistent with the changes at inoculation sites.

The differentially expressed genes (DEGs, at |log_2_FC| ≥ 1, *p* adjust < 0.05) were listed in Supplementary Materials Table S3.2–3.7. The KEGG enrichment analysis based on DEGs indicated a significant increase in flavonoid, flavone, and flavonol biosynthesis but a decrease in plant-pathogen interaction, phenylpropanoid, and isoflavonoid biosynthesis in local ‘ScPzH’ compared to ‘mock’ tissues (Supplementary Materials Figure S5A). For local ‘ScGyH2′ compared to ‘mock’ tissues, glutathione metabolism, sesquiterpenoid, and triterpenoid terpenoid backbone biosynthesis were up-regulated. Phenylpropanoid biosynthesis, fatty acid elongation, plant hormone signal transduction, plant-pathogen interaction, cutin, suberine, and wax biosynthesis were down-regulated (Supplementary Materials Figure S5B). Compared to ScPzH, ScGyH2 induced the levels of glutathione metabolism, terpenoid backbone, sesquiterpenoid, and triterpenoid biosynthesis but reduced the expression of photosynthesis, fatty acid elongation-related genes (Supplementary Materials Figure S5C). The GSEA (Gene Set Enrichment Analysis) based on gene expression in the local tissues exhibited consistent enrichment and was shown in Supplementary Materials Table S3.8. Meanwhile, the KEGG enrichment of systemic tissues generally indicated that similar patterns occurred in a local increase of flavonoid biosynthesis in systemic ‘ScPzH’; up-regulation of glutathione metabolism, terpenoid backbone biosynthesis, and down-regulation of fatty acid elongation in systemic ‘ScGyH2′; suppression of plant-pathogen interaction in both systemic ‘ScPzH’ and ‘ScGyH2′, but all performed on a smaller scale where relatively fewer functional genes were involved (Supplementary Materials Figure S6).

The infection of *Psa*, especially the high-pathogenicity strain, significantly re-programmed the host’s gene expression. Firstly, more salicylic acid (SA) signaling related genes, including NPR4 (Achn176311, Achn209911), PR1 (Achn240741, Achn146991, Achn253311), WRKY40 (Achn329191) and WRKY70 (Achn140751, Achn287671, Achn062591, Achn258601, Achn147941) were up-regulated in local ‘ScGyH2′ compared to ‘ScPzH’. However, ScGyH2 inoculated ‘Hongyang’ displayed more serious infection than ScPzH inoculated stems. Instead, genes involved in plant-pathogen interaction were down-regulated as shown in KEGG enrichment (Supplementary Materials Figures S5 and S6), and presented as the expression heatmap in Supplementary Materials Figure S7. Especially, the Ca^2+^ signaling pathway genes were found to be down-regulated in both ‘ScPzH’ and ‘ScGyH2′, including genes encoding CNGC, calcium-transporting ATPase, CaM/CML, CDPK (20 and 34 genes down-regulated in local ‘ScPzH’ and ‘ScGyH2′, respectively), and thus RBOH, WRKY22/33/29 that function downstream of the pathway (Table 2). Such suppression was also observed in the lateral indirect inoculation sites of systemic ‘ScPzH’ and ‘ScGyH2′, especially for ‘ScGyH2′ (Supplementary Materials Table S3.6). Furthermore, another significant expression suppression in local ‘ScGyH2′ presented in fatty acid elongation (19 genes encoding 3-ketoacyl-CoA synthase and very-long-chain 3-oxoacyl-CoA reductase, Supplementary Materials Figure S8 and Table 2), and cutin, suberine, wax biosynthesis (13 genes encoding P450-dependent fatty acid omega-hydroxylase, fatty acyl-CoA reductase, omega-hydroxypalmitate O-feruloyl transferase and alcohol-forming fatty acyl-CoA reductase, Table 2). Again, the remarkable down-regulation was confirmed in systemic ‘ScGyH2′. These genes’ suppression might affect secondary metabolism and plant immunity of ‘Hongyang’, considering the roles of cutin, suberine, and wax in pathogenic defense and their close association with long chain fatty acid.

From co-expression network analysis (WGCNA, Supplementary Materials Figure S9) of the RNA-seq data, the significant hub genes (Supplementary Materials Figure S10A), such as calcium-dependent protein kinase 1 (Achn255871), calcium-binding protein PBP1 (Achn176951), mitogen-activated protein kinase (Achn082251), linoleate lipoxygenase (Achn123641), and down-regulated transcription factors (Supplementary Materials Figure S10B), including fatty acid biosynthesis regulator WRINKLED 1 (Achn044161), WRKY29/22 (Achn132821, Achn278571, Achn275661), were further illustrated as candidates worth focusing on.

### 3.4. Validation of Gene Expression in Psa-Inoculated ‘Hongyang’

**Figure 5:**
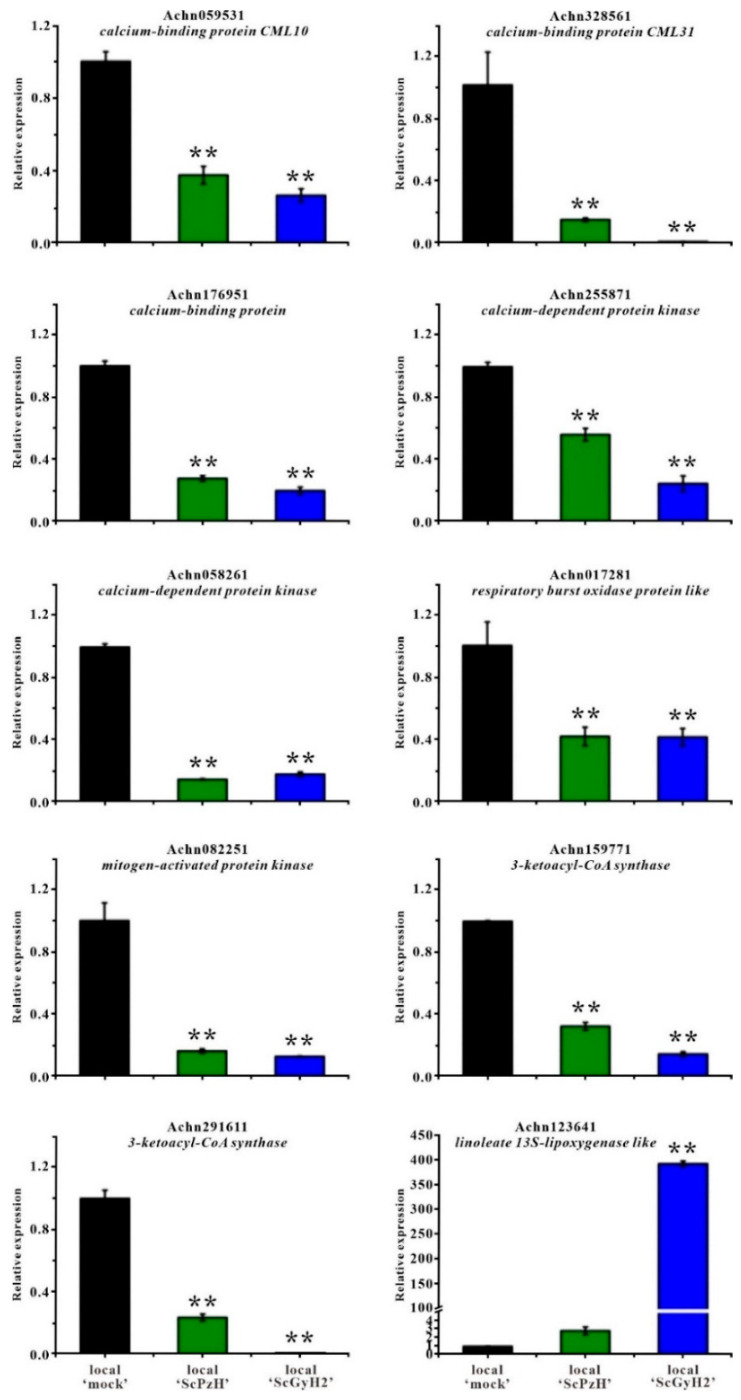
Validation of gene expressions in *Psa*-inoculated ‘Hongyang’ by RT-qPCR. Ten differentially expressed kiwifruit genes in transcriptomic analysis, encoding calcium-binding protein CML10 (Achn059531), calcium-binding protein CML31 (Achn328561), calcium-binding protein (Achn176951), calcium-dependent protein kinase (Achn255871, Achn058261), respiratory burst oxidase protein like (Achn017281), mitogen-activated protein kinase (Achn082251), 3-ketoacyl-CoA synthase (Achn159771, Achn291611) and linoleate 13S-lipoxygenase like (Achn123641), were determined for RT-qPCR in mock, ScPzH, or ScGyH2 inoculated stem tissues at inoculation sites (local ‘mock’, local ‘ScPzH and local ‘ScGyH2′, respectively) at 14 dpi. Significant differences compared with ‘mock’ were analyzed using Student’s *t*-test: ** *p* < 0.01.

To verify the expression profiles obtained from the RNA-seq data, ten differentially expressed genes (seven genes presented in Table 2 for their down-regulation upon *Psa* inoculation, the other three genes analyzed as hubs shown in Supplementary Materials Figure S10A) were randomly selected for real-time qPCR analysis using the primers listed in Supplementary Materials Table S1. As shown in Figure 5, six genes of the Ca^2+^ signaling pathway, *calcium-binding protein CML10* (Achn059531), *calcium-binding protein CML31* (Achn328561), *calcium-binding protein* (Achn176951), *calcium-dependent protein kinase* (Achn255871, Achn058261), and *respiratory burst oxidase protein* (Achn017281), were significantly down-regulated in ScPzH and ScGyH2 inoculated ‘Hongyang’ stems compared with the mock tissues at 14 dpi, as well as the gene of *mitogen-activated protein kinase* (Achn082251) exhibited the suppression of signaling transduction in *Psa*-inoculated tissues, especially in high-pathogenicity ScGyH2 infected ‘Hongyang’. Similar to RNA-seq analysis, the RT-qPCR determination also indicated remarkable suppression of the genes encoding 3-ketoacyl-CoA synthase (Achn159771, Achn291611) in the fatty acid elongation pathway. In addition, the abundance of *linoleate 13S-lipoxygenase* (Achn123641) showed slight and drastic increases, respectively, in local ‘ScPzH’ and ‘ScGyH2′, suggesting activation of unsaturated fatty acid metabolism. All the RT-qPCR determined genes confirmed consistent expression profiles in the RNA-seq analysis. The Ca^2+^ signaling transduction and fatty acid elongation-related genes were down-regulated in both low-pathogenicity ScPzH and high-pathogenicity ScGyH2 inoculated ‘Hongyang’, which was more obvious in serious infected ‘ScGyH2′ tissues except Achn058261 and Achn017281.

### 3.5. New Features in Psa-‘Hongyang’ Interactions

Taken together, the transcriptomic performance of infected ‘Hongyang’ reflected the interactions between kiwifruit and *Psa* variants. Consistent with previous reports [31,32,33,34], the SA signaling components, including NPR4, PR1, thaumatin-like protein, WRKY40, and WRKY70, were up-regulated in *Psa*-infected ‘Hongyang’ tissues, especially in seriously diseased ‘ScGyH2′ stems (Figure 4, Supplementary Materials Table S3). However, the asynchrony between SA pathway activation and disease prevention in the host plant suggests that SA-mediated defense, one of the important common responses of kiwifruit to *Psa*, is ineffective in rendering full resistance in ‘Hongyang’ [35]. Alternatively, the susceptibility of ‘Hongyang’ to *Psa* might be attributed to, at least partially, the down-regulation of plant genes controlling fatty acid elongation, cutin/suberine/wax biosynthesis, and calcium signal transduction (Table 2, Figure 5 and Supplementary Materials Figures S5–S8). The suppression was found in both ScPzH and ScGyH2 inoculated tissues but more serious in ScGyH2-infected stems, suggesting common molecular mechanisms utilized by *Psa* strains and their close correlation with host susceptibility. The Ca^2+^ signaling pathway plays an important role in plant defense, in which transient cytosolic Ca^2+^ elevations are sensed by CaM/CML/CDPK to further regulate ion channels, such as CNGC, Ca^2+^-ATPases, and their target proteins RBOH and WRKY [36]. This pathway was significantly suppressed in *Psa*-inoculated ‘Hongyang’ (Table 2). Although *Psa* biovar 3 is normally pathogenic to *Actinidia* species, some genotypes, such as *A. eriantha* and *A. latifolia*, are considerably resistant to *Psa* [20,37,38]. Similar resistance was observed in *A. eriantha* cv. ‘Huate’ against ScGyH2 (Supplementary Materials Figure S11). The up-regulation of Ca^2+^ signaling in resistant ‘Huate’, contrasting to the down-regulation in susceptible ‘Hongyang’ [34], added to the evidence of the role of Ca^2+^ transduction in *Psa* resistance. Moreover, the suppression of fatty acid elongation in *Psa*-infected ‘Hongyang’ (Supplementary Materials Figure S8) provides a novel point for this susceptibility. Long-chain fatty acid is involved in cutin, suberine, and wax (pathogenic defenders) biosynthesis, and a reduced level of unsaturated fatty acid enhances plant resistance to pathogens [39], emphasizing fatty acid metabolism as a part of *Psa*-kiwifruit interactions.

From the aforementioned comparative analysis, genomic with transcriptomic, it’s reasonable to assume that some unascertained *Psa* effectors interrupt host defense by interacting with their targets in these pathways to snatch a priority for pathogen colonization. In our previous work [3], the genome analysis of ‘Hongyang’ annotated fewer NBS-LRR genes (96) and a larger number of pattern-recognition receptors (261) than other plant species, implying the function of ancient innate PAMP-triggered immunity in *A*. *chinensis* against pathogens, while specific effector-triggered immunity may not be under strong selection pressure, presumably due to fewer pathogens adapted to kiwifruit before its domestication and thus extensive planting. In current transcriptomic analysis, several important effector-triggered immunity-associated genes, including RIN4 (RPM1-interacting protein 4, Achn228021), RAR1 (required for *Mla12* resistance, Achn010951), and SAG101 (senescence-associated carboxylesterase 101, Achn380061) [40,41,42], were up-regulated in ScGyH2-infected tissues but not in ScPzH-infected tissues (Supplementary Materials Table S3.4), suggesting effector-triggered immunity may be activated by high-pathogenicity *Psa* to develop new “players” to counteract virulent effectors in the further resistance evolution of this crop.

## 4. Conclusions

*P*. *syringae* pv. *actinidiae* is a successful pathogen to host crop kiwifruit (*Actinidia* spp.) considering its sudden emergence and rapid expansion, which brought *Psa*-kiwifruit interactions under follow-up investigation as a rare recent case for its origin, evolution, and improvement. In the present work, ten *Psa* strains were isolated from susceptible *A*. *chinensis* cv. ‘Hongyang’ for pathogenicity determination and genome sequencing to reveal the pathogenicity-associated loci of *Psa*, and thus the transcriptomic responses of ‘Hongyang’, locally and systemically, to *Psa* strains with differential pathogenicity. The resulting multifaceted analyses provide new insights into *Psa*-kiwifruit interactions and potential targets in signaling transduction and fatty acid mechanism for crop disease resistance improvements.

## Figures and Tables

**Table 1:** Genomic analysis of the *Psa* strains.

	ScDjyH (2016)	AhHfH (2017)	ScBcH (2017)	ScGyH1 (2017)	ScGyH2 (2017)	ScMcH (2017)	ScPzH (2017)	ScYaH (2018)	ScYbH (2018)	HbEsH (2019)
Genome size (bp)	6,219,076	6,206,124	6,063,125	6,156,719	6,145,566	6,157,061	6,078,771	6,170,143	6,264,163	6,255,125
CDS	5706	6035	5731	5676	5617	5679	5575	5728	6107	5607
Carbohydrate-active enzyme	127	127	127	126	125	126	125	126	128	129
Secondary metabolism cluster	8	7	7	7	8	7	8	8	7	7
Transporter	1039	1052	1044	1045	1039	1042	1036	1026	1047	1039
Drug-resistance	361	366	368	367	363	364	361	363	368	364
CRISPR-Cas	6	10	11	20	39	9	15	13	25	23
Virulence factor	684	693	684	694	691	693	684	682	694	692
Pathogen-host interaction	964	967	960	964	957	964	955	961	970	958
Secretory system	77	77	77	77	76	76	72	75	77	74
Secretory protein	361	369	348	375	324	376	348	345	354	308
Genomic island	2	9	10	5	6	4	1	6	7	5
Prophage	3	2	1	1	1	1	2	2	2	2

**Table 2:** Down-regulated genes in the Ca^2+^ signaling pathway, fatty acid elongation, cutin, suberine, and wax biosynthesis in local *Psa*-inoculated tissues.

Pathway	Local ‘Mock’ vs. Local ‘ScPzH’	Local ‘Mock’ vs. Local ‘ScGyH2′
Ca^2+^ signaling pathway		
CNGC (cyclic nucleotide-gated ion channel)		Achn162021, Achn176821, Achn293571, Achn165181, Achn289381
calcium-transporting ATPase	Achn012851, Achn373261, Achn370491, Achn275611, Achn030411	Achn012851, Achn373261, Achn370491, Achn275611, Achn030431, Achn378021, Achn378031
CaM/CML (calmodulin/calmodulin-like protein)	Achn059531, Achn089421, Achn014601, Achn089411, Achn328561, Achn136351, Achn235431, Achn030401, Achn039201, Achn067991	Achn059531, Achn089421, Achn014601, Achn089411, Achn328561, Achn136351, Achn235431, Achn030401, Achn039201, Achn328551, Achn039181, Achn235441, Achn237651, Achn327821, Achn328551
CDPK (calcium-dependent protein kinase)	Achn382671, Achn255871, Achn022761, Achn058261, Achn045801	Achn382671, Achn255871, Achn022761, Achn058261, Achn069421, Achn341081, Achn386651
RBOH (respiratory burst oxidase homolog)	Achn017281, Achn108551	Achn017281, Achn213151, Achn052291, Achn167921
WRKY22/33/29	Achn275661, Achn287861, Achn175001	Achn275661, Achn287861, Achn175001, Achn278571, Achn132821
fatty acid elongation		
3-ketoacyl-CoA synthase	Achn291611, Achn159771, Achn374091, Achn311381, Achn331621	Achn291611, Achn159771, Achn374091, Achn311381, Achn331621, Achn221911, Achn054161, Achn030011, Achn387451, Achn322471, Achn007461, Achn232681, Achn060641, Achn091941, Achn168451, Achn172321, Achn104701, Achn330241
very-long-chain 3-oxoacyl-CoA reductase		Achn350941
cutin, suberine, and wax biosynthesis		
P450-dependent fatty acid omega-hydroxylase	Achn018501, Achn017821, Achn018511	Achn018501, Achn017821, Achn018511, Achn380671, Achn119641, Achn098691, Achn336411, Achn195921
fatty acyl-CoA reductase		Achn185231
omega-hydroxypalmitate O-feruloyl transferase		Achn016941, Achn348111
alcohol-forming fatty acyl-CoA reductase		Achn005071, Achn305621

## Data Availability

The statement: The DNA and RNA sequencing data deposited in Sequence Read Archive (SRA) of NCBI for availability.

## Supplementary Materials

The supporting information can be downloaded at: https://www.mdpi.com/article/10.3390/ijms23179743/s1.
