# Supplementary material for: Genomic Variation and Host Interaction among Pseudomonas syringae pv. actinidiae Strains in Actinidia chinensis ‘Hongyang’
Source: Int J Mol Sci. 2022 Aug 28;23(17):9743. doi: 10.3390/ijms23179743 (PMC9456109; doi:10.3390/ijms23179743)
Supplement: Supplementary file 1 [file ijms-23-09743-s001.zip › Supplementary Figures.pdf]

Genomic variation and host interaction among *Pseudomonas syringae* pv. *actinidiae* strains in *Actinidia chinensis* 'Hongyang'

Yu Zhou[a†], Shengxiong Huang[a†], Wei Tang[b], Zhongqiu Wu[a], Siqi Sun[c], Yaqiong Qiu[a], Hongtao Wang[a], Xue Chen[a], Xiaofeng Tang[a], Fangming Xiao[d], Yongsheng Liu[b], Xiangli Niu[a]*

[a] School of Food and Biological Engineering, Hefei University of Technology, Hefei, Anhui 230601, China

[b] School of Horticulture, Anhui Agricultural University, Hefei, Anhui 230036, China

[c] Anhui Jiaotianxiang Biological Technology, Xuancheng, Anhui 242099, China

[d] Department of Plant Sciences, University of Idaho, Moscow, Idaho 83844, USA

[†] These authors contributed equally to this work.

* Correspondence: niu_xiangli@163.com

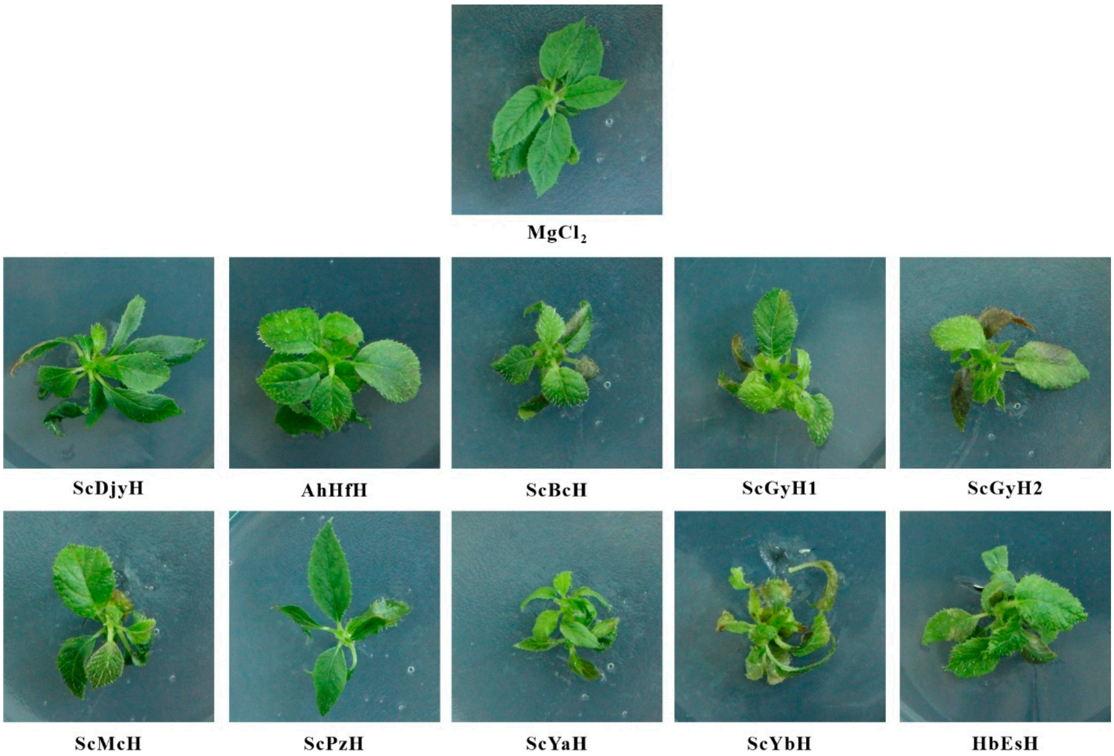

Supplementary Figure S1 Flood-inoculated ‘Hongyang’ at 12 dpi (days post-inoculation). The tissue-cultured ‘Hongyang’ explants were flood inoculated with *Psa* bacterial suspension (5×10^8 CFU) and grown at 16°C for 12 days. Sterile 10 mM $MgCl_2$ was used as a control.

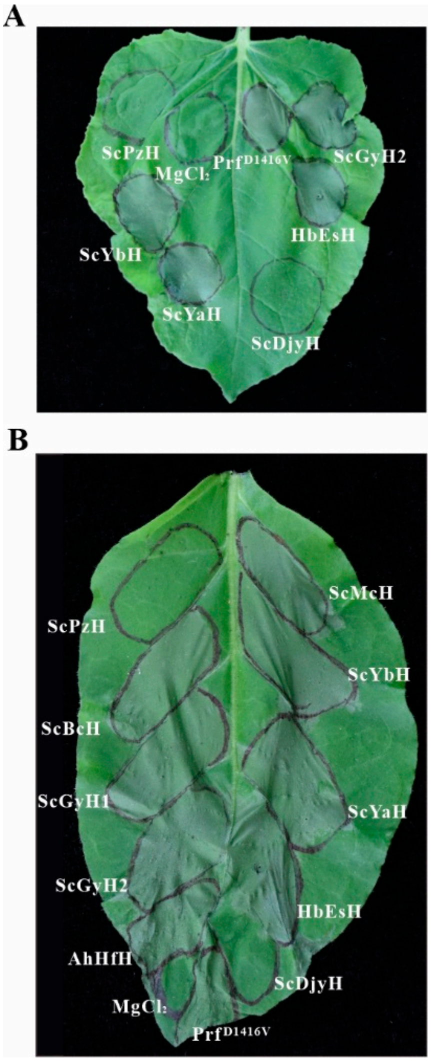

Supplementary Figure S2 Hypersensitivity response of *N. benthamiana* (A) and *N. tabacum* (B) elicited by the *Psa* strains. Leaves of 4-to 5-week-old tobacco plants were infiltrated with *Psa* suspension (5×10^8 CFU) for 72 h. Prf^{D1416V} or 10 mM $MgCl_2$ was injected as a positive or negative control, respectively.

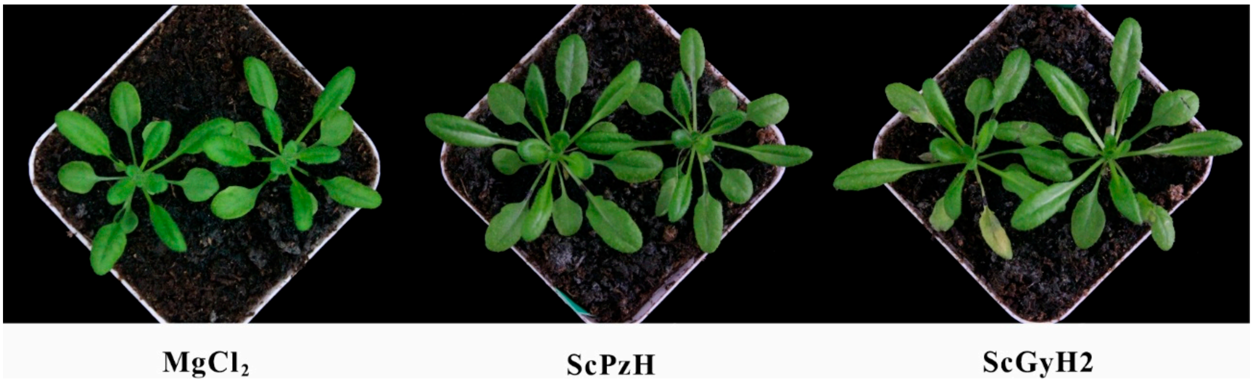

Supplementary Figure S3 Symptoms of *Arabidopsis thaliana* plants at 4 dpi. Four-week-old Arabidopsis rosette leaves (2-3 leaves for each plant) were infiltrated with ScPzH or ScGyH2 (10^7 CFU) by hand-infiltration using 1-mL syringes without a needle, and 10 mM $MgCl_2$ was used for control infiltration.

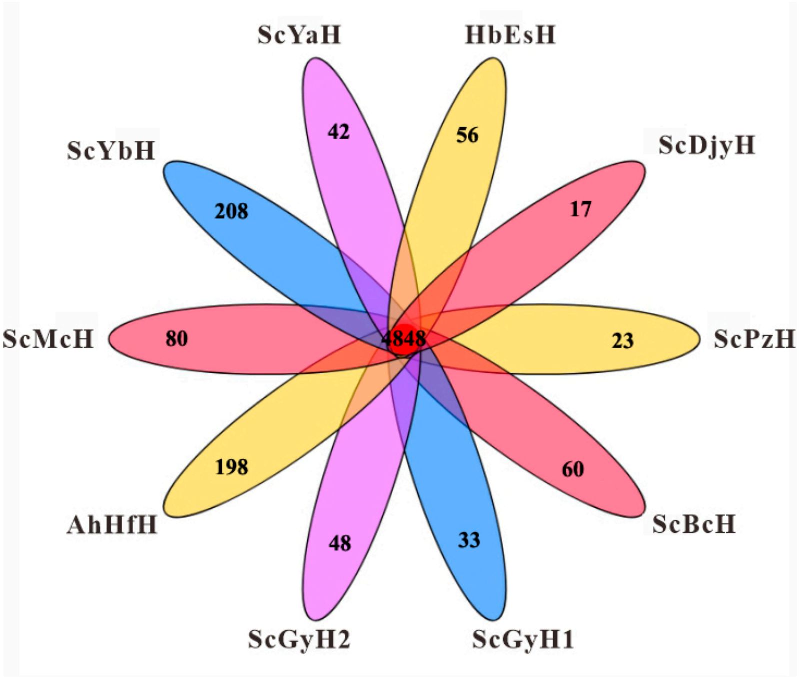

Supplementary Figure S4 The venn diagram with the numbers of core and specific genes among ten *Psa* strains.

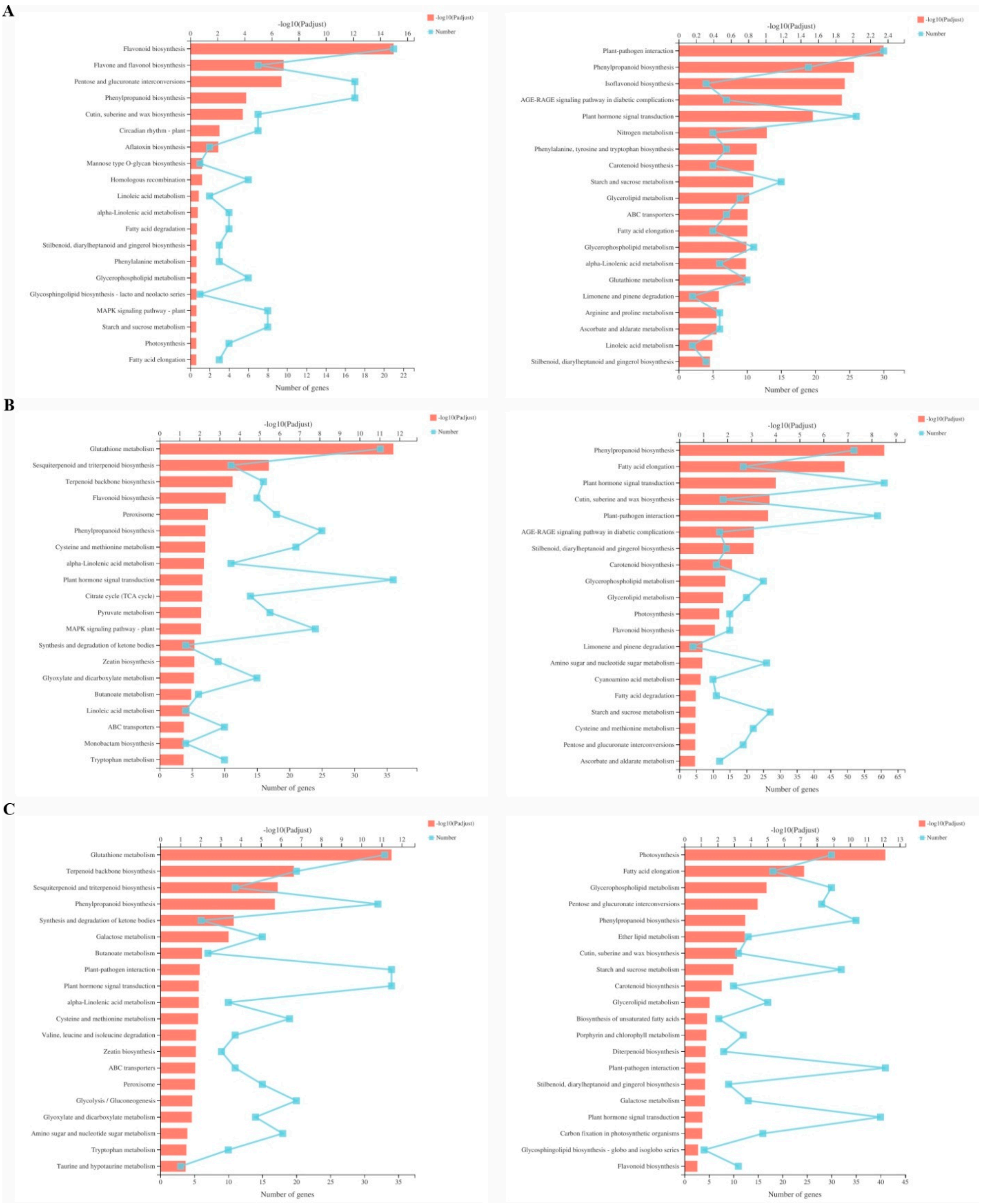

Supplementary Figure S5 The KEGG enrichment analysis of differentially expressed genes (DEGs) in ‘Hongyang’ tissues at

inoculation sites (local ‘mock’, local ‘ScPzH and local ‘ScGyH2’, respectively) as local ‘mock’ versus local ‘ScPzH’ (A), local ‘mock’ versus local ‘ScGyH2’ (B) and local ‘ScPzH’ versus local ‘ScGyH2’ (C). The up-regulated pathways were presented on the left, and the down-regulated pathways shown on the right of A, B and C.

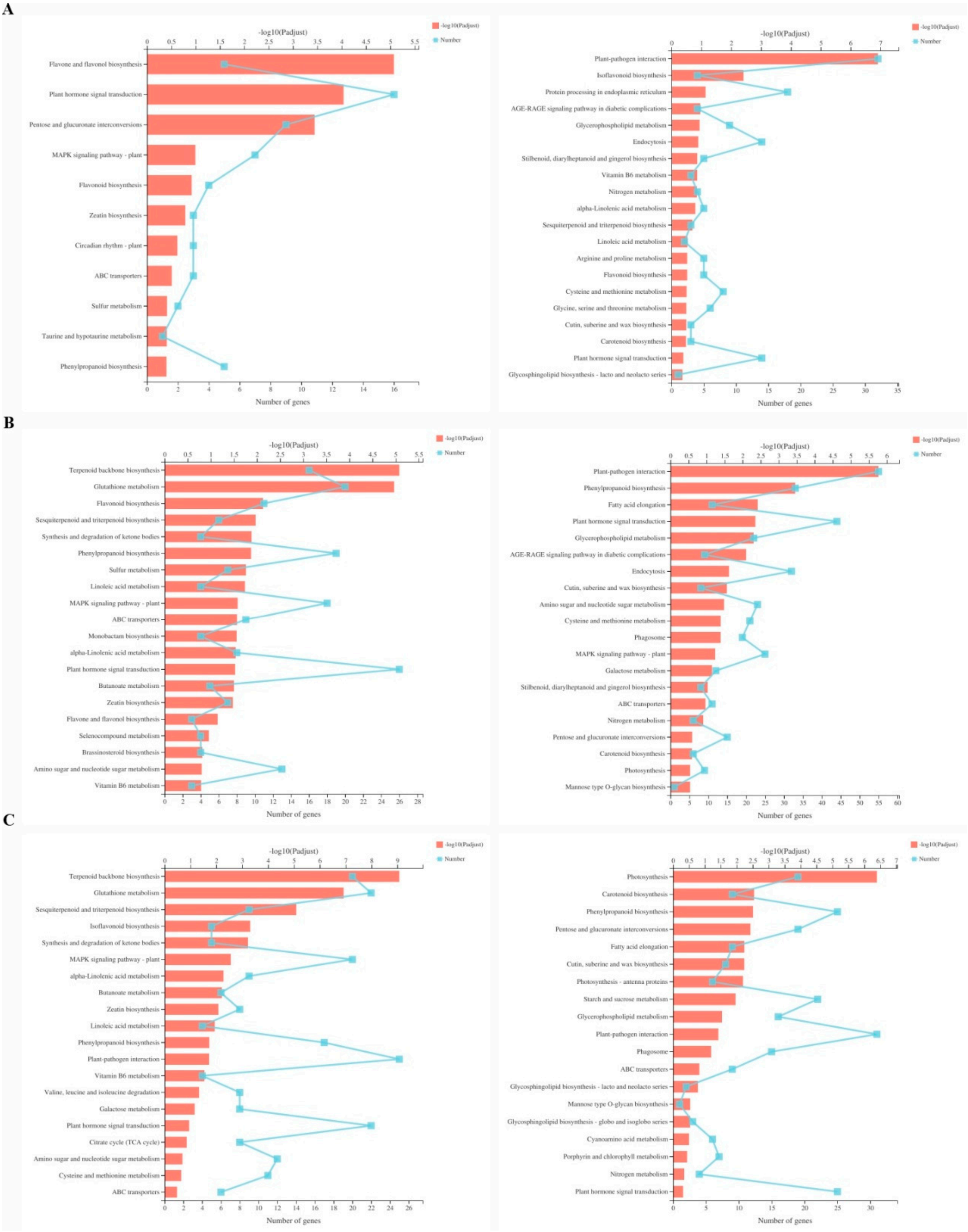

Supplementary Figure S6 The KEGG enrichment analysis of differentially expressed genes (DEGs) in 'Hongyang' tissues at adjacent

inoculation sites (systemic ‘mock’, systemic ‘ScPzH, systemic ‘ScGyH2’, respectively) as systemic ‘mock’ versus systemic ‘ScPzH’ (A), systemic ‘mock’ versus systemic ‘ScGyH2’ (B) and systemic ‘ScPzH’ versus systemic ‘ScGyH2’ (C). The up-regulated pathways were presented on the left, and the down-regulated pathways shown on the right of A, B and C.

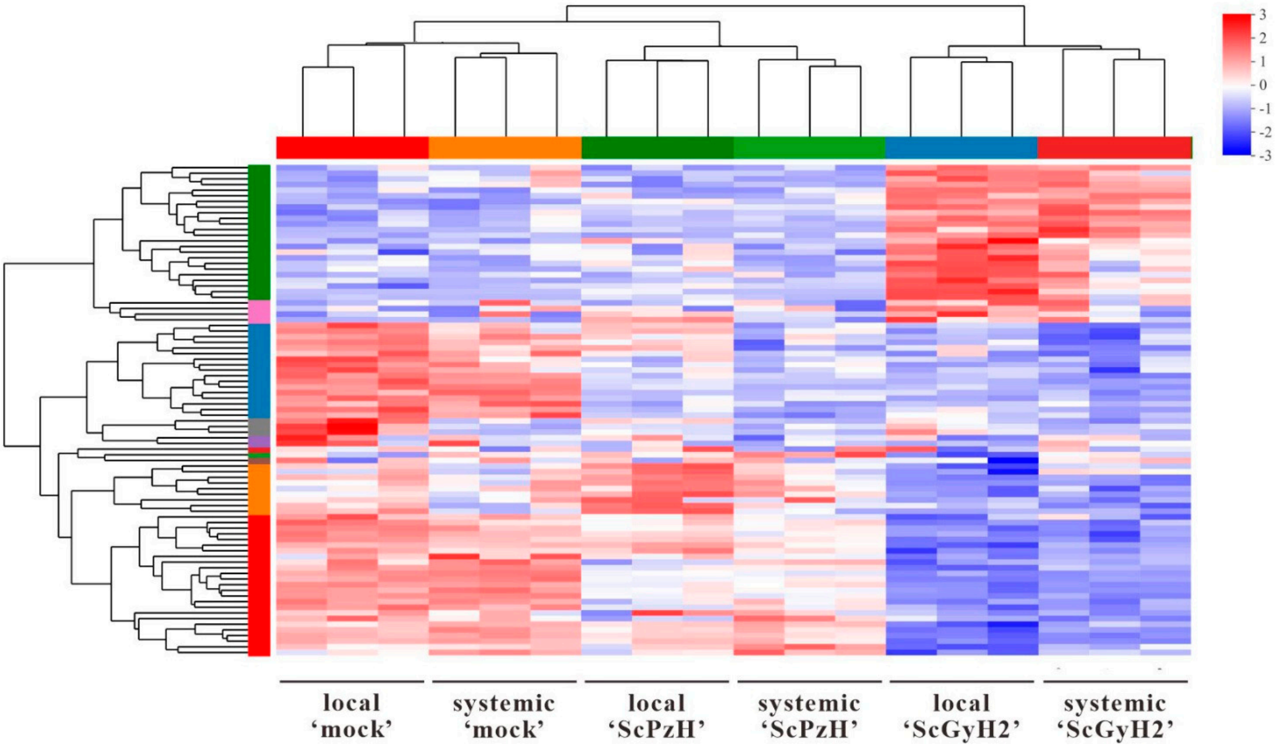

Supplementary Figure S7 Expression heatmap of the differentially expressed genes involved in plant-pathogen interaction in $MgCl_2$, ScPzH, or ScGyH2 inoculated tissues at inoculation sites (local 'mock', local 'ScPzH, local 'ScGyH2', respectively) and 2 cm above the inoculation sites (systemic 'mock', systemic 'ScPzH, systemic 'ScGyH2', respectively). Three biological replicates were included. The color scale indicates a relative fold-change in expression, where red color shows high expression and blue for low expression.

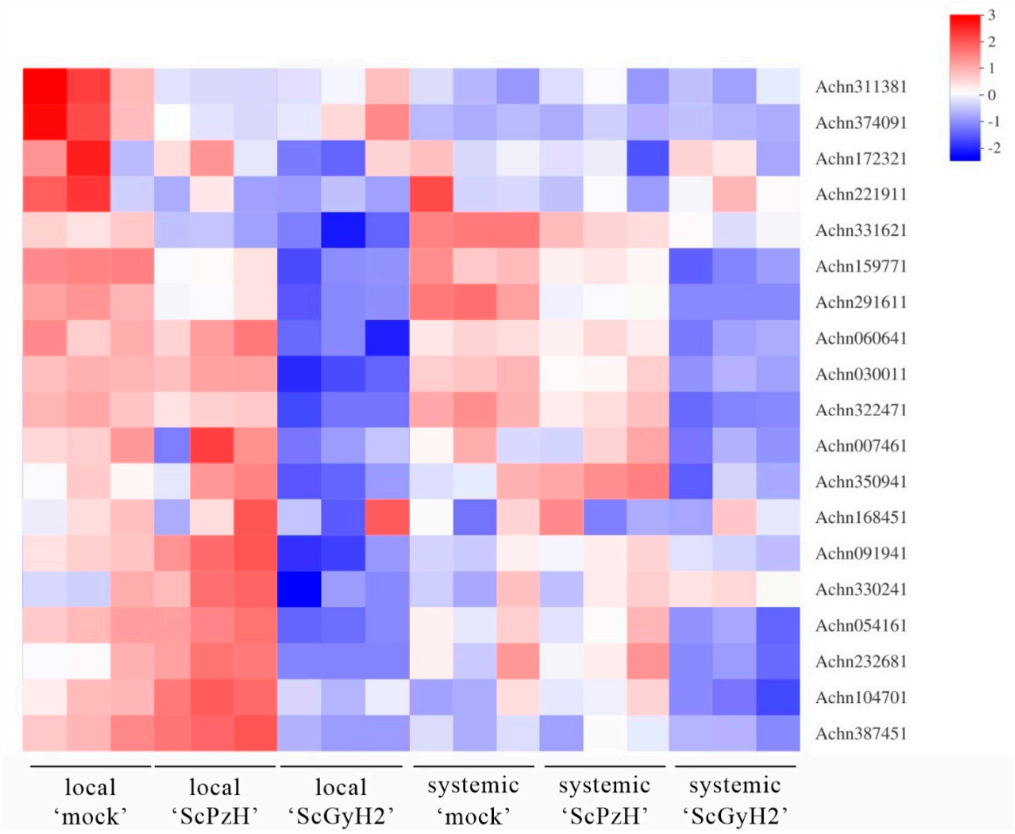

Supplementary Figure S8 Expression heatmap of the fatty acid elongation associated genes in $MgCl_2$, ScPzH, or ScGyH2 inoculated tissues at inoculation sites (local ‘mock’, local ‘ScPzH, local ‘ScGyH2’, respectively) and 2 cm above the inoculation sites (systemic ‘mock’, systemic ‘ScPzH, systemic ‘ScGyH2’, respectively). Three biological replicates were included. The color scale indicates a relative fold-change in expression, where red color shows high expression and blue for low expression.

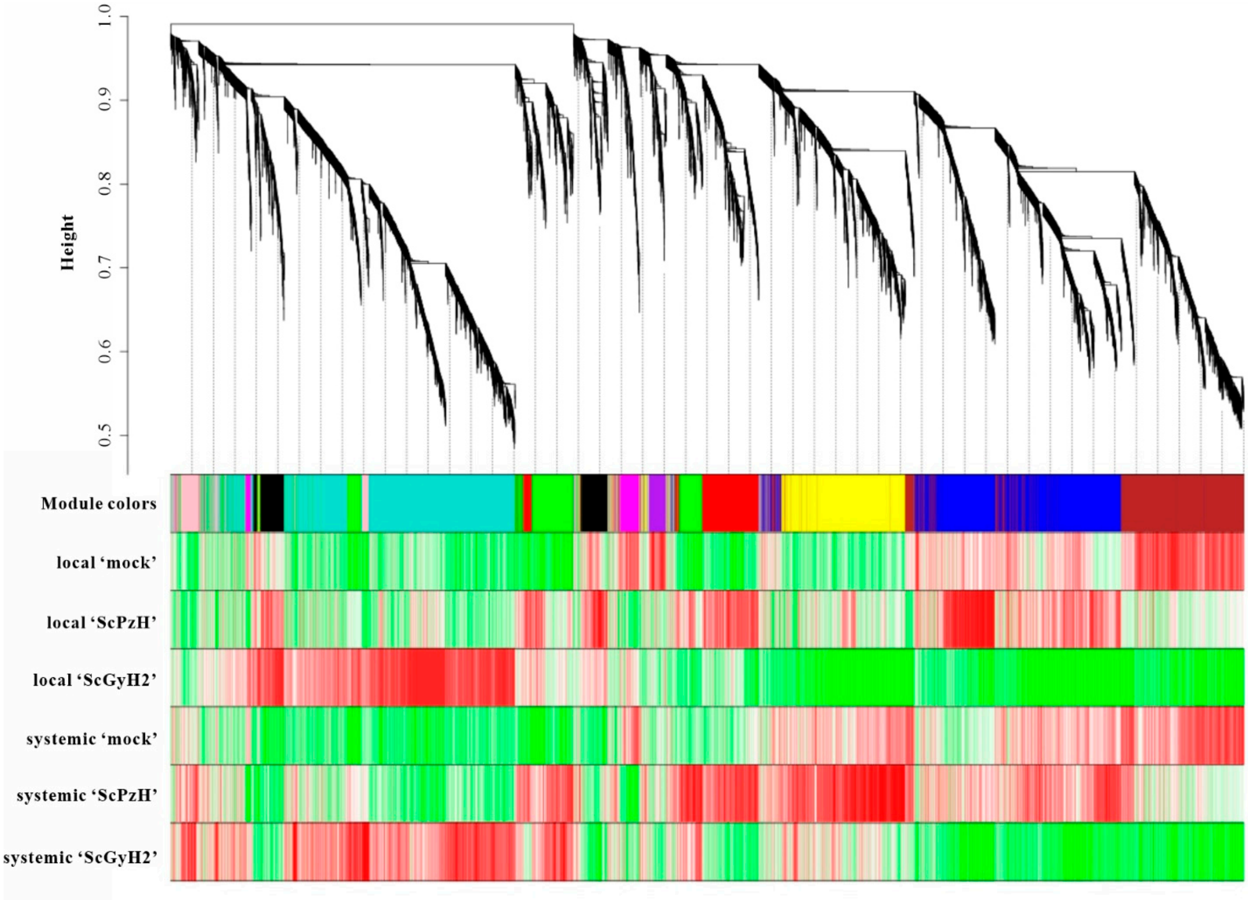

Supplementary Figure S9 The weighted gene co-expression network analysis (WGCNA) of the RNA-seq data from local ‘mock’, ‘ScPzH, ‘ScGyH2’ and systemic ‘mock’, ‘ScPzH, ‘ScGyH2’.

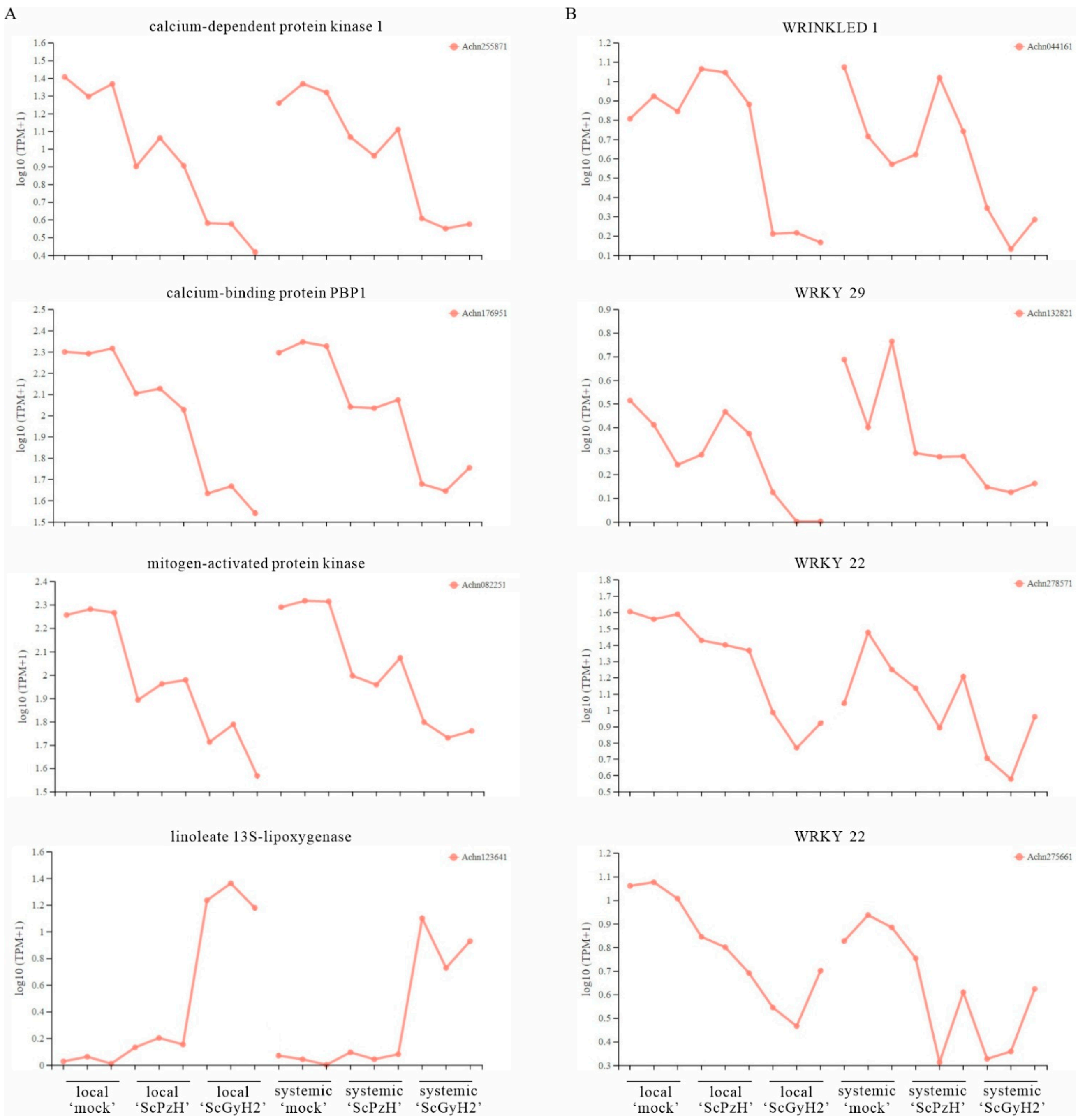

Supplementary Figure S10 The expression patterns of hub genes in WGCNA analysis (A) and down-regulated transcription factors (B) in RNA-seq quantification.

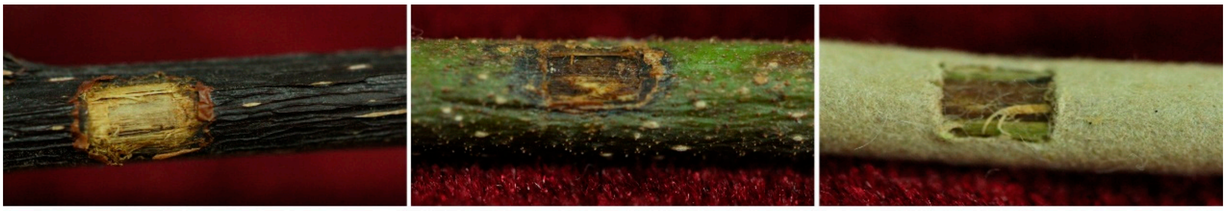

‘Hongyang’ ‘Hayward’ ‘Huate’

Supplementary Figure S11 Disease symptoms of *A. chinensis* cv. ‘Hongyang’, *A. deliciosa* cv. ‘Hayward’ and *A. eriantha* cv. ‘Huate’ at 15 dpi. The detached twigs of ‘Hongyang’, ‘Hayward’ and ‘Huate’ were wound-inoculated with ScGyH2 (10^9 CFU).
